# Supplementary material for: From sequence to enzyme mechanism using multi-label machine learning
Source: BMC Bioinformatics. 2014 May 19;15:150. doi: 10.1186/1471-2105-15-150 (PMC4229970; doi:10.1186/1471-2105-15-150)
Supplement: Additional file 2 — Java code of ml2db. Additional file ml2db_code.tar.gz contains the Java source code to run the multi-label machine learning experiments and save the results to database. The code’s Javadoc is included. [file 1471-2105-15-150-S2.zip › additional file 2/ml2db/ml2db/doc/main/ml2db/DbManagers.html]

DbManagers


JavaScript is disabled on your browser.


- Overview
- Package
- Class
- Use
- Tree
- Deprecated
- Index
- Help

- Prev Class
- Next Class

- Frames
- No Frames

- All Classes

- Summary:
- Nested |
- Field |
- Constr |
- Method

- Detail:
- Field |
- Constr |
- Method


main.ml2db

## Class DbManagers

- java.lang.Object
- - main.ml2db.DbManagers

- All Implemented Interfaces:
  :   java.io.Serializable

  ---

    

  ```
  public class DbManagers
  extends java.lang.Object
  implements java.io.Serializable
  ```

  Stores a database connection to the databases containing: 1. the data 2. the
  arff records and 3. where results should be written. Not all classes will
  need all three, so they are allowed to be null. For example, a MulanArff
  class will need only the data database as input and the arff database as
  output, but not the database for machine learning results.

  Version:
  :   28 Jul 2011

  Author:
  :   Luna De Ferrari ldf(at)st-andrews.ac.uk

  See Also:
  :   Serialized Form

- - ### Constructor Summary

    Constructors

    Constructor and Description

    `DbManagers(uk.ac.standrews.utils.main.database.DbManager dataDb,
    uk.ac.standrews.utils.main.database.DbManager arffDb,
    uk.ac.standrews.utils.main.database.DbManager mlResultsDb)`

    `DbManagers(java.lang.String dataDbPropsFile,
    java.lang.String arffDbManagerPropsFile,
    java.lang.String resultsDbManagerPropsFile)`
  - ### Method Summary

    Methods

    | Modifier and Type | Method and Description |
    | --- | --- |
    | `void` | `closeConnections()` Closes all three database connections |
    | `uk.ac.standrews.utils.main.database.DbManager` | `getArffDb()` |
    | `uk.ac.standrews.utils.main.database.DbManager` | `getDataDb()` |
    | `uk.ac.standrews.utils.main.database.DbManager` | `getMlResultsDb()` |
    | `static void` | `main(java.lang.String[] args)` |
    | `void` | `refreshConnection()` |

    - ### Methods inherited from class java.lang.Object

      `equals, getClass, hashCode, notify, notifyAll, toString, wait, wait, wait`

- - ### Constructor Detail


    - #### DbManagers

      ```
      public DbManagers(uk.ac.standrews.utils.main.database.DbManager dataDb,
                uk.ac.standrews.utils.main.database.DbManager arffDb,
                uk.ac.standrews.utils.main.database.DbManager mlResultsDb)
      ```


    - #### DbManagers

      ```
      public DbManagers(java.lang.String dataDbPropsFile,
                java.lang.String arffDbManagerPropsFile,
                java.lang.String resultsDbManagerPropsFile)
      ```
  - ### Method Detail


    - #### closeConnections

      ```
      public void closeConnections()
      ```

      Closes all three database connections


    - #### getArffDb

      ```
      public uk.ac.standrews.utils.main.database.DbManager getArffDb()
      ```


    - #### getDataDb

      ```
      public uk.ac.standrews.utils.main.database.DbManager getDataDb()
      ```


    - #### getMlResultsDb

      ```
      public uk.ac.standrews.utils.main.database.DbManager getMlResultsDb()
      ```


    - #### refreshConnection

      ```
      public void refreshConnection()
      ```


    - #### main

      ```
      public static void main(java.lang.String[] args)
      ```


- Overview
- Package
- Class
- Use
- Tree
- Deprecated
- Index
- Help

- Prev Class
- Next Class

- Frames
- No Frames

- All Classes

- Summary:
- Nested |
- Field |
- Constr |
- Method

- Detail:
- Field |
- Constr |
- Method
